# Collagen induction of immune cells in the mammary glands during pregnancy

**DOI:** 10.1152/physiolgenomics.00098.2023

**Published:** 2023-11-13

**Authors:** Karen Yamaguchi, Jun Nakayama, Tomofumi Yamamoto, Kentaro Semba, Tatsuo Shirota, Yusuke Yamamoto

**Affiliations:** ^1^Laboratory of Integrative Oncology, National Cancer Center Research Institute, Tokyo, Japan; ^2^Department of Oral and Maxillofacial Surgery, Showa University School of Dentistry, Tokyo, Japan; ^3^Department of Oncogenesis and Growth Regulation, Research Institute, Osaka International Cancer Institute, Osaka, Japan; ^4^Department of Life Science and Medical Bioscience, School of Advanced Science and Engineering, Waseda University, Tokyo, Japan; ^5^Department of Molecular and Cellular Medicine, Institute of Medical Science, Tokyo Medical University, Tokyo, Japan; ^6^Division of Biological Chemistry and Biologicals, National Institute of Health Sciences, Kanagawa, Japan; ^7^Translational Research Center, Fukushima Medical University, Fukushima, Japan

**Keywords:** collagen, immune cells, mammary gland, pregnancy, single-cell RNA-sequencing

## Abstract

The mammary glands are dynamic tissues affected by pregnancy-related hormones during the pregnancy-lactation cycle. Collagen production and its dynamics are essential to the remodeling of the mammary glands. Alterations of the mammary microenvironment and stromal cells during the pregnancy-lactation cycle are important for understanding the physiology of the mammary glands and the development of breast tumors. In this study, we performed an evaluation of collagen dynamics in the mammary fat pad during the pregnancy-lactation cycle. Reanalysis of single-cell RNA-sequencing (scRNA-Seq) data showed the ectopic collagen expression in the immune cells and cell-cell interactions for collagens with single-cell resolution. The scRNA-Seq data showed that type I and type III collagen were produced not only by stromal fibroblasts but also by lymphoid and myeloid cell types in the pregnancy phase. Furthermore, the total cell-cell interaction score for collagen interactions was dramatically increased in the pregnancy tissue. The data presented in this study provide evidence that immune cells contribute, at least in part, to mammary collagen dynamics. Our findings suggest that immune cells, including lymphoid and myeloid cells, might be supportive members of the extracellular matrix orchestration in the pregnancy-lactation cycle of the mammary glands.

**NEW & NOTEWORTHY** Our study evaluated mammary gland collagen dynamics during the pregnancy-lactation cycle using single-cell RNA-sequencing data. We found ectopic collagen expression in immune cells and an increase in collagen interactions during pregnancy. Type I and type III collagen were produced by lymphoid, myeloid, and stromal fibroblast cells during pregnancy. These findings suggest that immune cells, including lymphoid and myeloid cells, play a crucial role in supporting the extracellular matrix in mammary glands during pregnancy-lactation cycles.

## Graphical abstract

**Figure:**
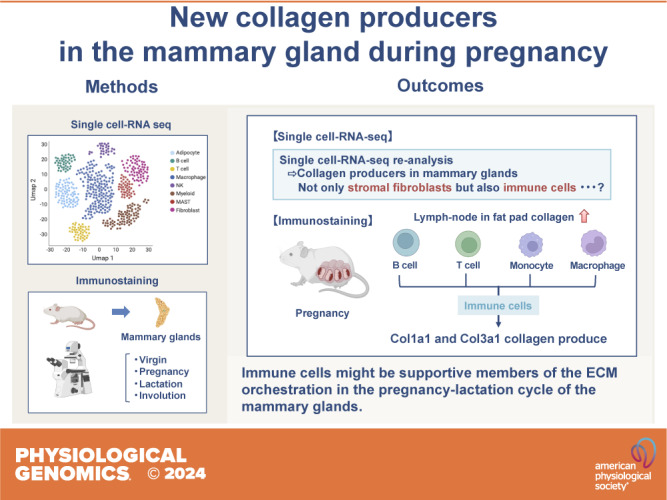

## INTRODUCTION

The mammary glands are dynamic tissues affected by pregnancy-associated hormones during the pregnancy-lactation cycle (1). The structure of the mammary glands is dramatically remodeled during pregnancy, lactation, and involution of the mammary glands. Remodeling of the mammary glands during the pregnancy-lactation cycle involves diverse cell types and the extracellular matrix (ECM) (2).

ECM remodeling is an important event during the pregnancy-lactation cycle (3). Type I and type III collagen make up the majority of collagen present in the body and have regulatory effects on cell adhesion, migration, proliferation, wound healing, ductal branching of mammary epithelial cells, and β-casein secretion (4). Collagen production and its dynamics are essential to the remodeling of the mammary glands. In general, stromal fibroblasts are the main producers of collagen in this tissue (5). Epithelial cell-stromal fibroblast interactions using Shank-associated RH domain-interacting protein (SHARPIN; also known as SIPL1) regulate the normal invasive mammary gland branching morphogenesis in an epithelial cell-extrinsic manner by controlling the organization of the stromal ECM (6). On the other hand, immune cells, such as T helper cells, regulate the mammary gland development by cytokine signaling in the pregnancy-lactation cycle (7). Macrophages in the involution phase remodel the ECM microenvironment via degradation by collagen phagocytosis and endocytosis (8, 9). In addition, altered tumorigenicity at distinct stages of postpregnancy mammary involution correlates with immune cells in the mammary tissues (10). Alterations of the mammary microenvironment and stromal cells during the pregnancy-lactation cycle are important for understanding the physiology of the mammary gland and the development of breast tumors.

In this study, we performed the evaluation of collagen dynamics in the mammary fat pad during the pregnancy-lactation cycle. Reanalysis of single-cell RNA-sequencing (scRNA-Seq) data showed the ectopic collagen expression in the immune cells, validated by histological observation and immunofluorescence. Also, cell-cell interactions (CCIs) for collagens were remarkably elevated during pregnancy.

## MATERIALS AND METHODS

### Reanalysis of Public Databases

We reused reliable scRNA-Seq data in the mouse cell atlas (11). Digital gene expression (DGE) files modified as a removal bath effect were downloaded from the figshare website (MCA DGE Data, https://figshare.com/s/865e694ad06d5857db4b). The scRNA-Seq analysis, including log normalization, principal component analysis, clustering, uniform manifold approximation and projection (UMAP) analysis, and extraction of signature genes in the subpopulations, was performed in *R* software with Seurat version 4 (12). We subset the expression data of “Mammary gland.Virgin,” “MammaryGland.Pregnancy,” “MammaryGland.Lactation,” and “MammaryGland.Involution” for reanalysis from the mouse cell atlas. Integration of single-cell data was performed by Harmony algorithms (13).

### Animal Study and Immunohistochemical Staining

All animal experiments were approved by the Animal Committee of Waseda University (2020-A067 and 2021-A074) and conformed to Animal Research: Reporting of In Vivo Experiments (ARRIVE) guidelines and the National Institutes of Health (NIH) *Guide for the Care and Use of Laboratory Animals* (NIH Pub. No. 8023, Revised 1978).

Mammary glands were harvested from BALB/c CrSlc mice (virgin 12 wk old, pregnancy day 13/12 wk old, lactation day 2/12 wk old, and involution day 2/12 wk old, Sankyo Labo Service, Tokyo, Japan). *Sample 1* and *sample 2* were harvested from independent mice. Each mammary gland was fixed in 4% paraformaldehyde overnight and embedded in paraffin. Mammary gland sections were dewaxed with xylene and rehydrated with ethanol (100%–70%). Hematoxylin and eosin staining, Masson’s trichrome staining, and Azan staining were performed by Kotobiken Medical Laboratories (Kotobiken Laboratories, Tokyo, Japan). Antigen retrieval was performed by boiling the specimens in Immunosaver (Nissin EM, Tokyo, Japan) diluted 1:200 for 45 min at 98°C. The sections were permeabilized with 0.1% Triton X-100 (Sigma Aldrich Japan, Tokyo, Japan) for 15 min. After being blocked with Dako blocking reagent for 30 min, sections were incubated with primary antibodies overnight at 4°C in a humidified box. Primary antibody for Col3a1 (ab184993, Abcam, Cambridge, UK), Col1a1 (No. 72026S, Cell Signaling Technology), CD3 (GTX628462, Gene Tex), CD11b (NB600-1327, Novus Biologicals, Centennial, CO), CD19 (GTX83282, Gene Tex), and vimentin (ab8978, Abcam) were used for immunofluorescence. Secondary antibodies conjugated to the following fluorophores were used: Hoechst 33342 Trihydrochloride Trihydrate (H3570, Thermo Fisher Scientific), Alexa Fluor 594 (Col3a1 and Col1a1) (A11012, Thermo Fisher Scientific), and Alexa Fluor 488 (CD3, CD11b, CD19, and vimentin) (A11001, Thermo Fisher Scientific). Images were captured using a BZ-X700 fluorescence microscope (Keyence, Osaka, Japan). Quantification was conducted with ImageJ software based on area size (*n* = 3 or 4 samples).

### Cell-Cell Interaction Analysis

CCI analysis was performed using the interaction database of Bader’s laboratory from Toronto University (https://baderlab.org/CellCellInteractions#Download_Data) in *R*, described as a previous research (14–16). The collagen (col family gene) interaction was extracted from Bader’s interaction list and used for the CCI analysis.

### Code Validity

The source code of transcriptome analysis in *R* is available on GitHub (https://github.com/JunNakayama/Reanalysis-of-MouseCellAtlas).

## RESULTS

### Single Cell Transcriptomic Analysis of Mammary Glands in Mice

**Figure 1.**
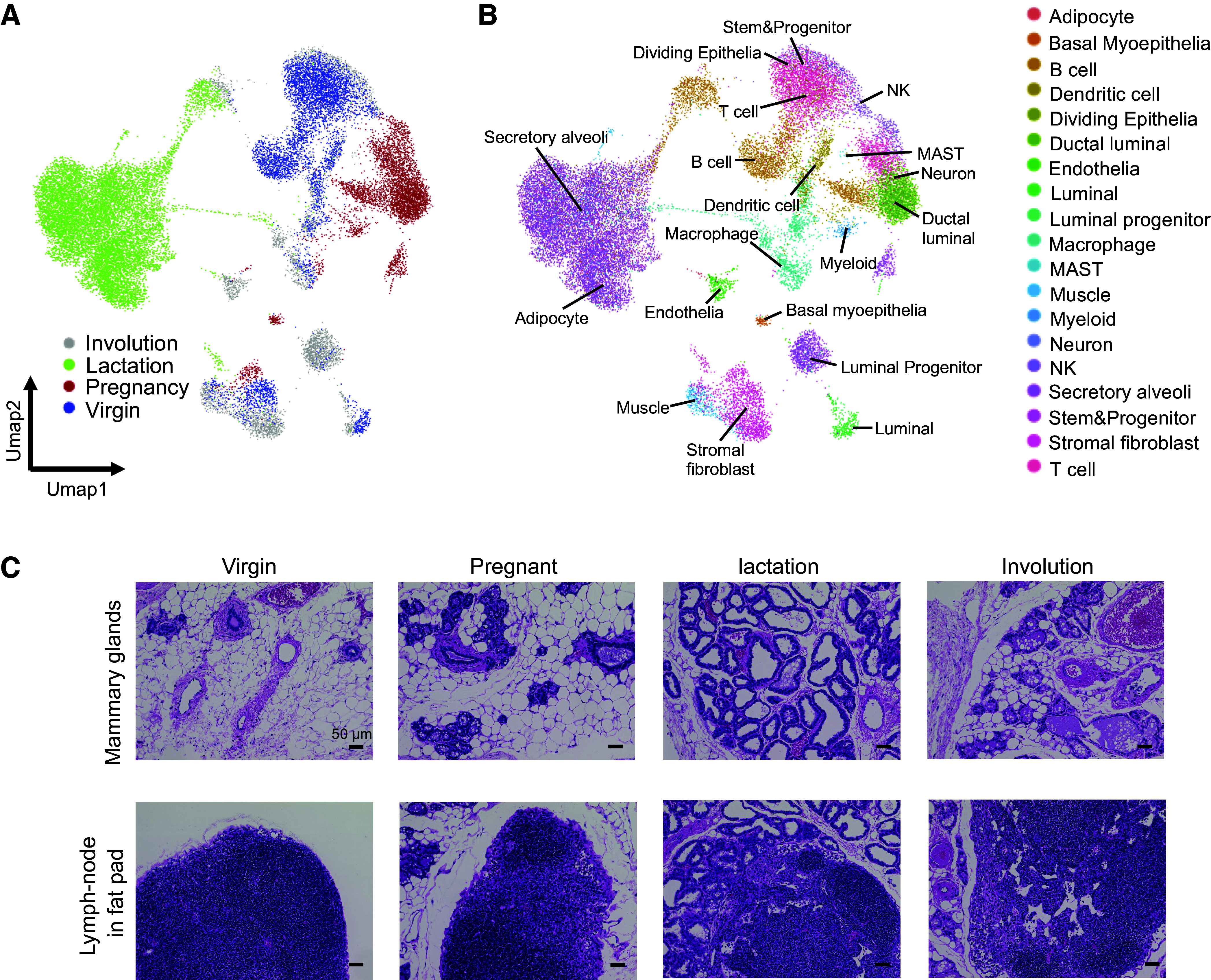
Immune cell populations of mammary glands during pregnancy. *A*: UMAP plot of scRNA-Seq of whole mammary glands (virgin, pregnancy, lactation, and involution). *B*: UMAP plot of scRNA-Seq with cell type information. *C*: hematoxylin and eosin staining of mammary gland tissues (virgin, pregnancy, lactation, and involution). *Top*: mammary glands; *bottom*: lymph nodes in the fat pad. Scale bar = 50 μm.

**Figure 2.**
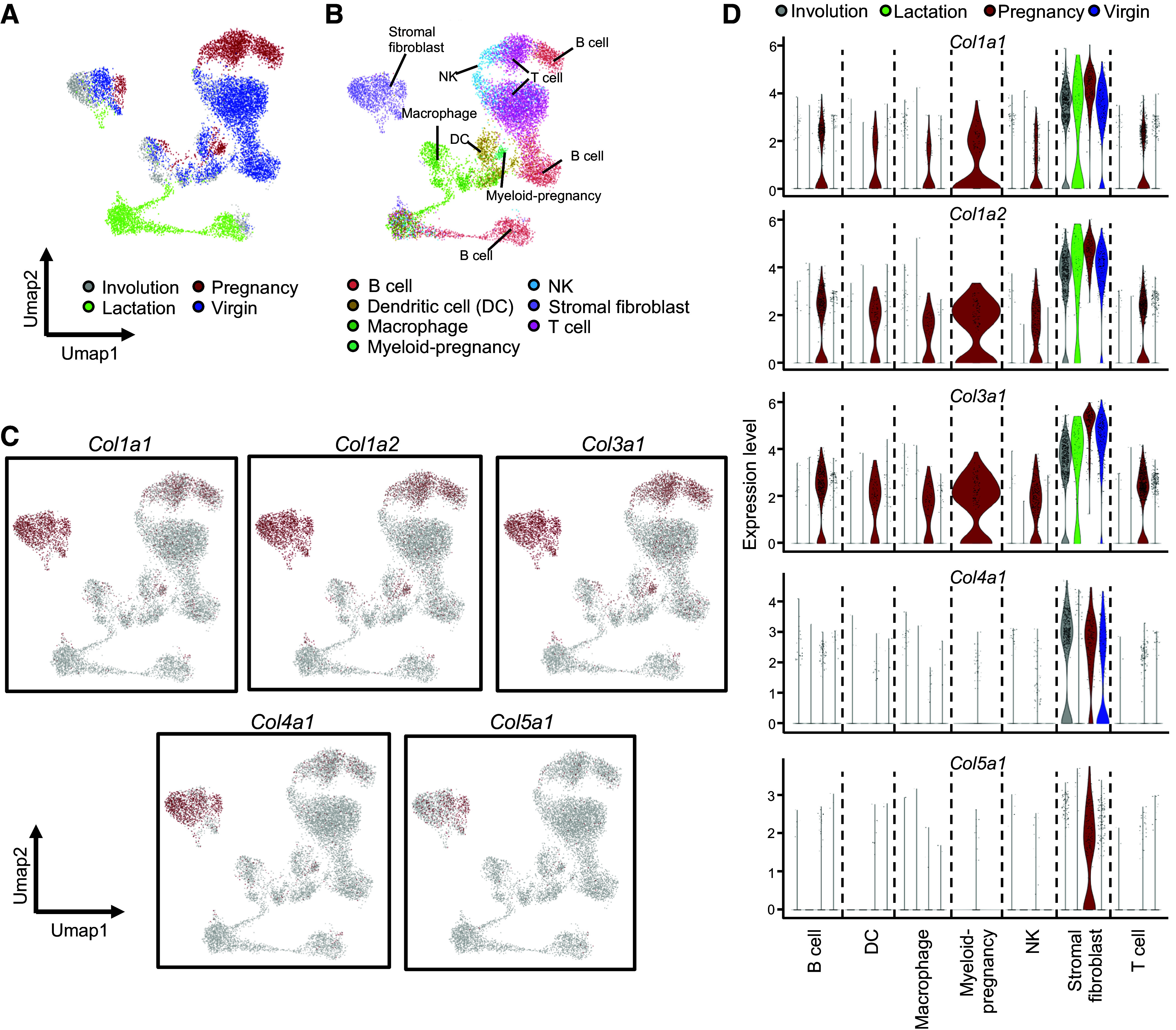
Ectopic collagen expression in immune cells at single-cell resolution. *A*: UMAP plot of scRNA-Seq data for immune cells and stromal cells in mouse mammary glands from a mouse cell atlas with sample state information. *B*: UMAP plot with cell type information. *C*: feature plots of collagen genes in scRNA-Seq with UMAP dimension. Collagen-expressing cells are shown in red. *D*: violin plots of collagen family genes across all phases.

To determine the collagen dynamics at single-cell resolution, we reanalyzed scRNA-Seq data from a mouse cell atlas by drop-seq, including data for mammary glands under virgin, pregnant, lactation, and involution conditions (Fig. 1, *A* and *B*, and Supplemental Table S1) (11). Mammary gland tissues showed drastic structure remodeling during the pregnancy-lactation cycle (Fig. 1*C*). Data for stromal cells and immune cells were extracted to identify microenvironmental effects across mammary gland remodeling (Fig. 2, *A* and *B*). Previously, fibrillar collagen types I, III, and V were found to make up the most of the total detected collagens in the mammary fat pad (17). We identified the expression of these collagen genes at single-cell resolution. Interestingly, type I and type III collagen were produced not only by stromal fibroblasts but also by immune cells such as lymphoid and myeloid cell types in the pregnancy phase (Fig. 2, *C* and *D*). Expression of *Col1a1*, *Col1a2*, and *Col3a1* was highly detected in both stromal fibroblasts and immune cells (Fig. 2*C*). Particularly, these expressions were restricted in the pregnancy phase (Fig. 2*D*). Type IV and type V collagens were supplied by only stromal fibroblasts (Fig. 2, *C* and *D*). Thus, our data suggested that collagen dynamics in the pregnancy-lactation cycle might be regulated by immune cells as well as stromal fibroblasts.

In addition, stromal fibroblasts produced type I and type III collagens during the entire pregnancy-lactation cycle; however, type V collagen was produced only in the pregnancy phase of the mammary glands (Fig. 2*D*). On the other hand, type IV collagen production from stromal fibroblasts decreased in the lactation phase (Fig. 2*D*). These results implied the possibility that collagen expression of immune cells during the pregnancy-lactation cycle might regulate lactation physiology, i.e., the development of mammary ducts and secretion of milk.

### Validation of Ectopic Expression of Collagens in Mammary Glands

**Figure 3.**
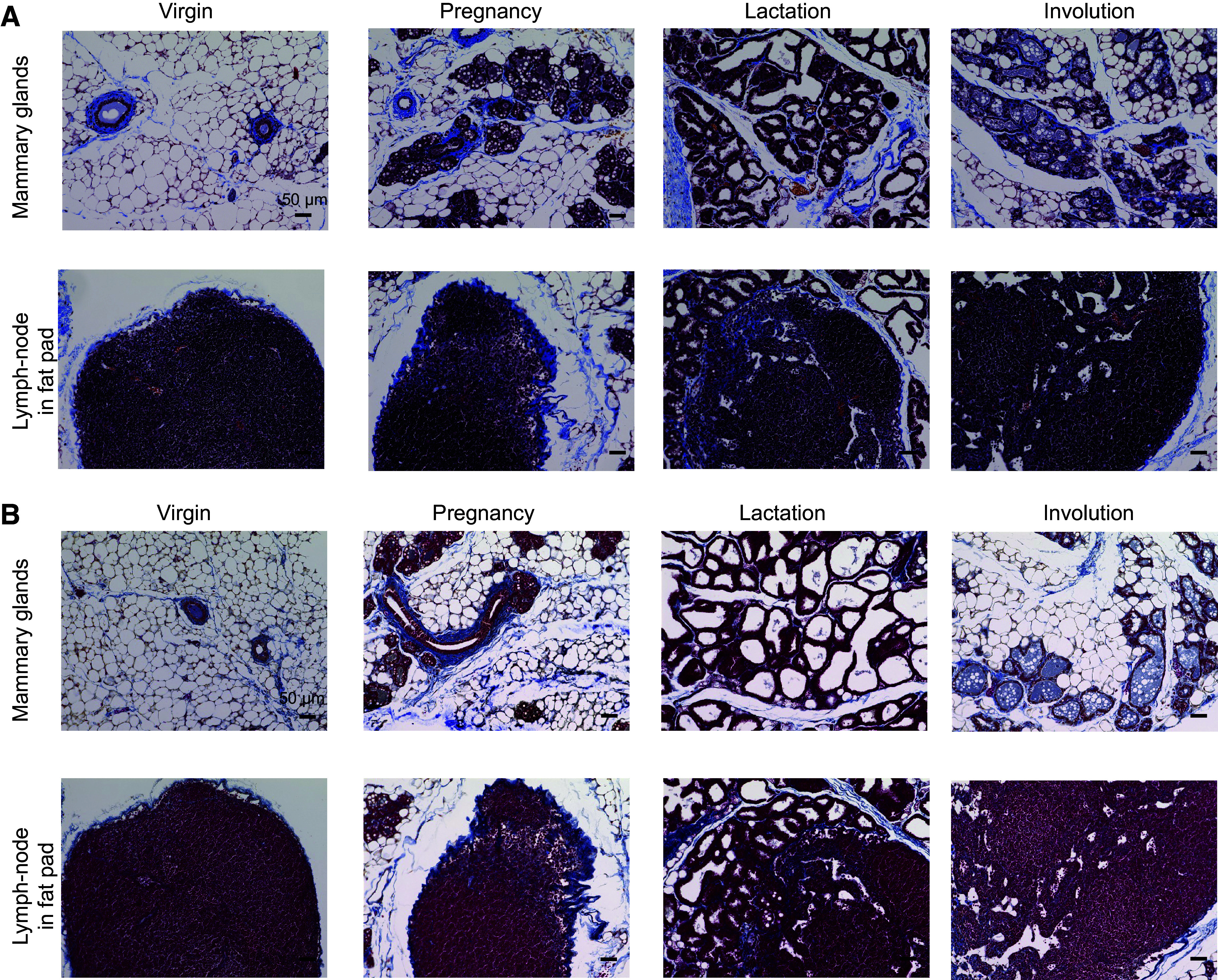
Histological analysis of collagen expression in mammary glands. *A*: Masson’s trichrome staining of mammary gland tissues (*top*) and lymph nodes in the fat pad (*bottom*). *B*: Azan staining of mammary gland tissues. *Top*: mammary glands; *bottom*: lymph nodes in the fat pad. Scale bar = 50 μm.

**Figure 4.**
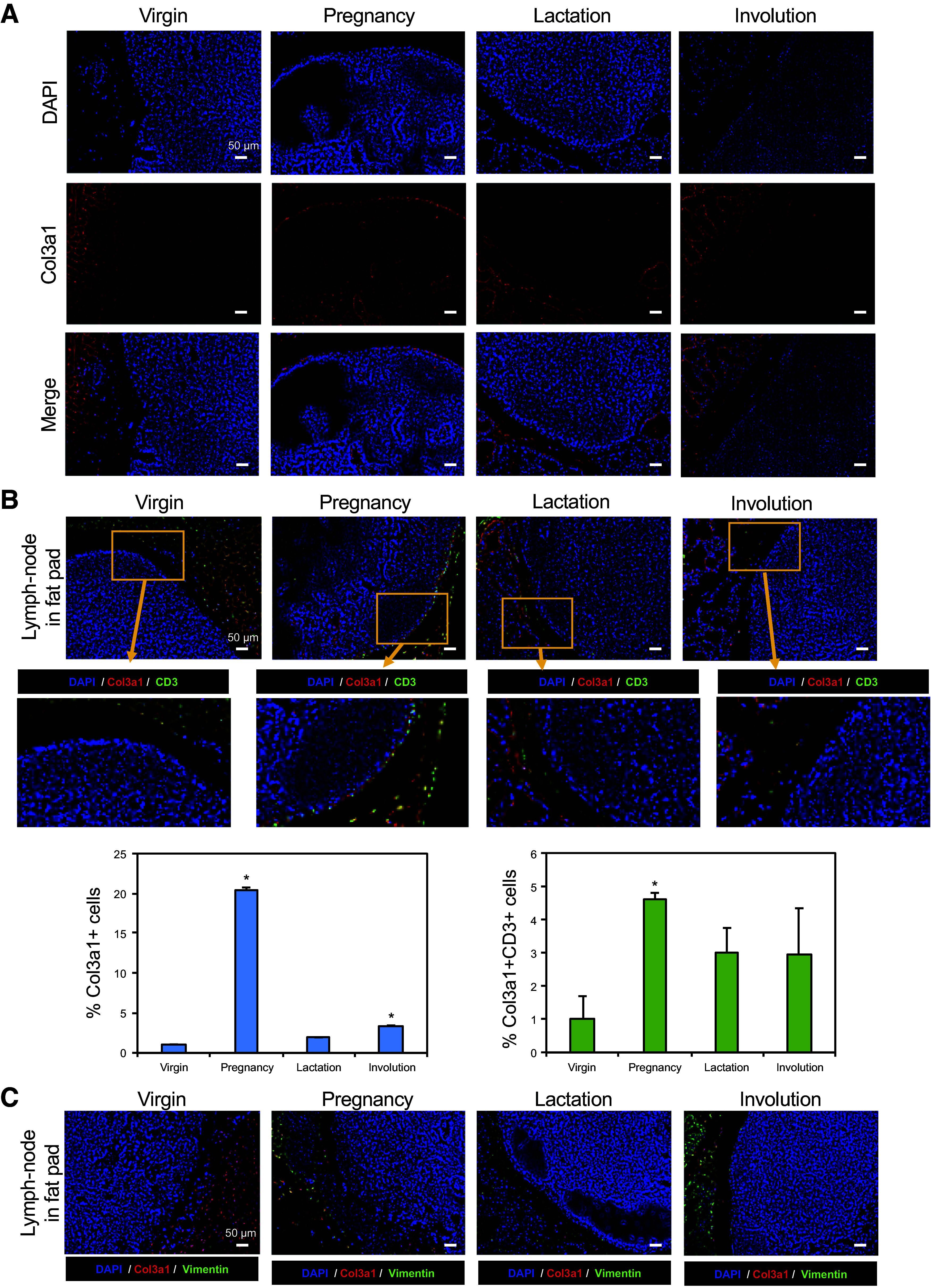
Immunofluorescence staining of collagen expression in lymph nodes in the fat pad. *A*: immunofluorescence staining of Col3a1 (red) and DAPI (blue). *B*: immunofluorescence staining of Col3a1 (red), CD3 (green), and DAPI (blue). The *left* graph shows the percentage of Col3a1 single-positive cells in mammary lymph nodes. *P < 0.05. The *right* graph shows the percentage of Col3a1^+^CD3^+^ cells in mammary lymph nodes. *P < 0.05. *C*: immunofluorescence staining of Col3a1 (red), vimentin (green), and DAPI (blue). Scale bar = 50 μm.

Next, we sought to histologically confirm the ectopic expression of collagens in the pregnancy phase. The histological analysis with hematoxylin and eosin, Masson’s trichrome, and Azan staining of the mammary gland showed that collagen and collagen-bound tissue increased during pregnancy (Fig. 1*C* and Fig. 3, *A* and *B*). Collagen fibers were strongly stained during the pregnancy-lactation cycle (Fig. 3, *A* and *B*, *top*). Ectopic collagens were also detected in lymph nodes of fat pads (Fig. 3, *A* and *B*, *bottom*). Immunostaining of mouse mammary glands was performed to confirm ectopic collagens. These data showed that Col3a1 levels dramatically increased in mammary tissue and fat pad lymph nodes during the pregnancy-lactation phases (Fig. 4*A* and Supplemental Fig. S1). We subsequently performed costaining of Col3a1 and CD3, a marker of T cells, to investigate the origin of the production of these collagens. We found Col3a1 and CD3 double-positive T cells in lymph nodes during the pregnancy phase (Fig. 4*B* and Supplemental Fig. S2). Approximately 4.6% of cells were found as Col3a1+/CD3+ T cells (Fig. 4*B*, *bottom right*). Also, ∼4.4% of Col3a1-positive cells coexpressed CD11b, a marker of monocytes, during the pregnancy phase (Supplemental Fig. S3). In addition to this, we also performed costaining of Col1a1 and CD19, a marker of B cells. Immunofluorescence showed Col1a1 and CD19 double-positive B cells (5.3%) in the lymph nodes during the pregnancy phase (Supplemental Fig. S4).

When Col3a1 was also costained with vimentin, a marker of fibroblasts, no overlap between Col3a1 and vimentin was observed in lymph nodes at any time (Fig. 4*C* and Supplemental Fig. S5), suggesting that Col3a1 in lymph nodes was not produced by stromal fibroblasts. Also, immunofluorescence of Col1a1 and vimentin confirmed almost no double-positive cells in the lymph nodes (Supplemental Fig. S6). These results suggest that collagen in the lymph nodes may be regulated by immune cells.

### Drastic Increase in Cell-Cell Interactions of the Collagen Network in the Pregnancy Phase

**Figure 5.**
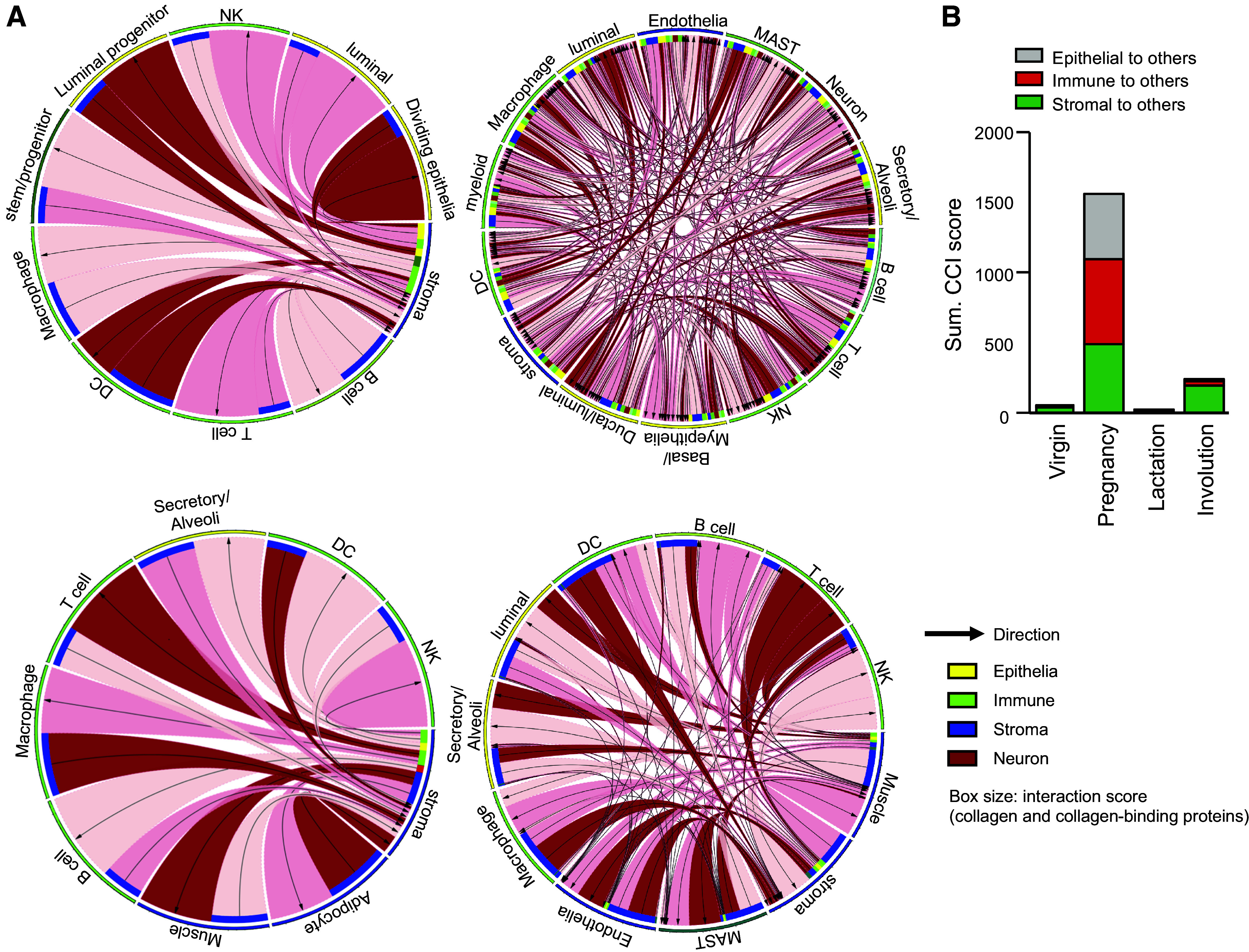
Cell-cell interaction (CCI) analysis with the collagen network. *A*: CCI analysis of mammary gland tissues using collagen genes and collagen-binding genes at single-cell resolution. A Circos plot showing collagen CCIs in the virgin, pregnancy, lactation, and involution states is shown. Arrows indicate “from collagens to collagen-binding proteins.” Yellow box: epithelia; green box: immune; blue box: stroma (including endothelia, muscle, and adipocyte); red box: neuron. Box size indicates interaction scores of collagens with collagen-binding proteins based on scRNA-Seq results. *B*: bar plot of the sum of CCI scores in the mammary glands.

Finally, using scRNA-Seq data of mammary glands, we performed CCI analysis to elucidate collagen networks. We extracted collagen and collagen-binding proteins from the mammary datasets to calculate the potency of CCI in all phases. Collagen interactions occurred primarily between stromal fibroblasts and other types of cells in the virgin, lactation, and involution states, but immune cells also actively interacted via type I and type III collagens in the pregnancy state (Fig. 5*A*). Also, frequencies of collagen interactions among cell types remarkably increased in the pregnancy state. The total CCI score for collagen interactions was dramatically increased in the pregnancy tissue (Fig. 5*B* and Supplemental Tables S2–S6). The involution phase also showed a higher CCI score for collagen interactions than the virgin and lactation phases.

## DISCUSSION

The present study elucidated collagen expression and dynamics in the mammary gland during pregnancy and lactation. Immune cells, mainly macrophages, are known to regulate collagen homeostasis through phagocytosis and degradation of collagen by endocytosis (8, 9) and may be involved in ECM generation to maintain homeostasis during the pregnancy-lactation cycle, as revealed by scRNA-Seq in this study.

Previous studies have reported that collagen gene mRNA is produced in mammary tissue during gestation and regression. During lactation, collagen production has been reported to decrease throughout the mammary fat pad (17). Our results support the dynamics of collagen during the pregnancy-lactation cycle. In addition, we identified the expression of collagen in immune cells during gestation. Previous studies have also reported that macrophages produce collagen in myocardial scar formation (18, 19). Thus, immune cells may have the potential to express collagen upon some stimulus associated with pregnancy. Our staining results showed increased collagen around and inside the lymph nodes in the mammary fat pad and found Col3a1^+^ T cells around the lymph nodes in the pregnancy phase. Collagen is important for maintaining the structure of lymph nodes and lymphatic vessels (20–22) and may be involved in the dramatic remodeling of mammary tissue.

The present study has a couple of limitations. The present data found a situation in which immune cells express collagen genes, but the physiological significance of this situation has not been fully elucidated. In particular, we could not show any evidence of ectopic collagen expression in the human mammary gland during pregnancy. If immune cells play an important role in producing collagen during the pregnancy state, it might be interesting to determine if autoimmune diseases are problematic for human mammary gland development and function. Further functional analysis is needed to assess the role of collagen dynamics in the mammary gland during pregnancy-lactation in human and mice. Another is regarding cell type classification in scRNA-Seq. As our reanalysis of scRNA-Seq across all phases of mouse mammary glands identified immune cells expressing collagen, we concluded that it was ectopic expression of collagen during pregnancy, but it may be a known cell type. For example, fibrocytes are known as collagen-producing monocyte-derived cells that also express hematopoietic markers (23). Further analysis of the molecular mechanism of this collagen expression is needed to understand the remodeling of the ECM by immune cells.

In summary, in this study, we identified collagen expression by immune cells in the mammary gland during the pregnancy phase. Our findings suggest that collagen dynamics in the mammary gland is regulated not only by stromal fibroblasts but also by immune cells. We also suggest that immune cells, including lymphocytes and myelocytes, may be supporting members of the ECM orchestration during gestation-lactation. Reanalysis of scRNA-Seq atlas data revealed new physiological phenomena in mammary gland biology.

## DATA AVAILABILITY

Data will be made available upon reasonable request.

## SUPPLEMENTAL DATA

10.6084/m9.figshare.24465985.v1 Supplemental Figs. S1–S6 and Supplemental Tables S1–S6: https://doi.org/10.6084/m9.figshare.24465985.v1.

## GRANTS

This work was supported by the Japan Society for the Promotion of Science (JSPS) KAKENHI [Grants 18K16269 and 21K15562: Grant-in-Aid for Early Career Scientist (to J.N.); Grant 20J01794: Grant in Aid for JSPS fellows (to J.N.); and Grant 21H02721: Grant-in-Aid for Scientific Research B (to Y.Y.)].

## DISCLOSURES

No conflicts of interest, financial or otherwise, are declared by the authors.

## AUTHOR CONTRIBUTIONS

J.N. and Y.Y. conceived and designed research; K.Y., J.N., and T.Y. performed experiments; K.Y., J.N., and T.Y. analyzed data; K.Y., J.N., T.Y., and Y.Y. interpreted results of experiments; K.Y., J.N., and T.Y. prepared figures; K.Y., J.N., and Y.Y .drafted manuscript; K.Y., J.N., and Y.Y. edited and revised manuscript; K.Y., J.N., T.Y., K.S., T.S., and Y.Y. approved final version of manuscript.

## References

[1] Colleluori G, Perugini J, Barbatelli G, Cinti S. Mammary gland adipocytes in lactation cycle, obesity and breast cancer. Rev Endocr Metab Disord 22: 241–255, 2021. doi:10.1007/s11154-021-09633-5. 33751362 PMC8087566

[2] Reed JR, Schwertfeger KL. Immune cell location and function during post-natal mammary gland development. J Mammary Gland Biol Neoplasia 15: 329–339, 2010. doi:10.1007/s10911-010-9188-7. 20730636 PMC4204476

[3] Schedin P, Keely PJ. Mammary gland ECM remodeling, stiffness, and mechanosignaling in normal development and tumor progression. Cold Spring Harb Perspect Biol 3: a003228, 2011. doi:10.1101/cshperspect.a003228. 20980442 PMC3003460

[4] Tsutsui S, Wakasa H, Tsugami Y, Suzuki T, Nishimura T, Kobayashi K. distinct expression patterns of fibrillar collagen types I, III, and V in association with mammary gland remodeling during pregnancy, lactation and weaning. J Mammary Gland Biol Neoplasia 25: 219–232, 2020. doi:10.1007/s10911-020-09457-0. 32915396

[5] Maller O, Martinson H, Schedin P. Extracellular matrix composition reveals complex and dynamic stromal-epithelial interactions in the mammary gland. J Mammary Gland Biol Neoplasia 15: 301–318, 2010. doi:10.1007/s10911-010-9189-6. 20811805

[6] Peuhu E, Kaukonen R, Lerche M, Saari M, Guzmán C, Rantakari P, De Franceschi N, Wärri A, Georgiadou M, Jacquemet G, Mattila E, Virtakoivu R, Liu Y, Attieh Y, Silva KA, Betz T, Sundberg JP, Salmi M, Deugnier MA, Eliceiri KW, Ivaska J. SHARPIN regulates collagen architecture and ductal outgrowth in the developing mouse mammary gland. EMBO J 36: 165–182, 2017. doi:10.15252/embj.201694387. 27974362 PMC5239997

[7] Watson CJ, Oliver CH, Khaled WT. Cytokine signalling in mammary gland development. J Reprod Immunol 88: 124–129, 2011. doi:10.1016/j.jri.2010.11.006. 21255846

[8] O'Brien J, Lyons T, Monks J, Lucia MS, Wilson RS, Hines L, Man YG, Borges V, Schedin P. Alternatively activated macrophages and collagen remodeling characterize the postpartum involuting mammary gland across species. Am J Pathol 176: 1241–1255, 2010. doi:10.2353/ajpath.2010.090735. 20110414 PMC2832146

[9] Jürgensen HJ, Van Putten S, Nørregaard KS, Bugge TH, Engelholm LH, Behrendt N, Madsen DH. Cellular uptake of collagens and implications for immune cell regulation in disease. Cell Mol Life Sci 77: 3161–3176, 2020. doi:10.1007/s00018-020-03481-3. 32100084 PMC11105017

[10] Hitchcock J, Hughes K, Pensa S, Lloyd-Lewis B, Watson CJ. The immune environment of the mammary gland fluctuates during post-lactational regression and correlates with tumour growth rate. Development 149: dev200162, 2022. doi:10.1242/dev.200162. 35420674 PMC9124574

[11] Han X, Wang R, Zhou Y, Fei L, Sun H, Lai S, Saadatpour A, Zhou Z, Chen H, Ye F, Huang D, Xu Y, Huang W, Jiang M, Jiang X, Mao J, Chen Y, Lu C, Xie J, Fang Q, Wang Y, Yue R, Li T, Huang H, Orkin SH, Yuan GC, Chen M, Guo G. Mapping the mouse cell atlas by microwell-seq. Cell 172: 1091–1107.e17, 2018 [Erratum in *Cell* 173: 1307, 2018]. doi:10.1016/j.cell.2018.02.001. 29474909

[12] Stuart T, Butler A, Hoffman P, Hafemeister C, Papalexi E, Mauck WM 3rd, Hao Y, Stoeckius M, Smibert P, Satija R. Comprehensive integration of single-cell data. Cell 177: 1888–1902.e21, 2019. doi:10.1016/j.cell.2019.05.031. 31178118 PMC6687398

[13] Korsunsky I, Millard N, Fan J, Slowikowski K, Zhang F, Wei K, Baglaenko Y, Brenner M, Loh PR, Raychaudhuri S. Fast, sensitive and accurate integration of single-cell data with Harmony. Nat Methods 16: 1289–1296, 2019. doi:10.1038/s41592-019-0619-0. 31740819 PMC6884693

[14] Nakayama J, Yamamoto Y. Cancer-prone phenotypes and gene expression heterogeneity at single-cell resolution in cigarette-smoking lungs. Cancer Res Commun 3: 2280–2291, 2023. doi:10.1158/2767-9764.CRC-23-0195. 37910161 PMC10637260

[15] Watanabe N, Fujita Y, Nakayama J, Mori Y, Kadota T, Hayashi Y, Shimomura I, Ohtsuka T, Okamoto K, Araya J, Kuwano K, Yamamoto Y. Anomalous epithelial variations and ectopic inflammatory response in chronic obstructive pulmonary disease. Am J Respir Cell Mol Biol 67: 708–719, 2022. doi:10.1165/rcmb.2021-0555OC. 36108172

[16] Tokura M, Nakayama J, Prieto-Vila M, Shiino S, Yoshida M, Yamamoto T, Watanabe N, Takayama S, Suzuki Y, Okamoto K, Ochiya T, Kohno T, Yatabe Y, Suto A, Yamamoto Y. Single-cell transcriptome profiling reveals intratumoral heterogeneity and molecular features of ductal carcinoma in situ. Cancer Res 82: 3236–3248, 2022. doi:10.1158/0008-5472.CAN-22-0090. 35852797

[17] Guo Q, Sun D, Barrett AS, Jindal S, Pennock ND, Conklin MW, Xia Z, Mitchell E, Samatham R, Mirza N, Jacques S, Weinmann S, Borges VF, Hansen KC, Schedin PJ. Mammary collagen is under reproductive control with implications for breast cancer. Matrix Biol 105: 104–126, 2022. doi:10.1016/j.matbio.2021.10.006. 34839002

[18] Lim GB. Macrophages produce collagen for myocardial scar formation. Nat Rev Cardiol 17: 267, 2020. doi:10.1038/s41569-020-0353-4. 32066868

[19] Simões FC, Cahill TJ, Kenyon A, Gavriouchkina D, Vieira JM, Sun X, Pezzolla D, Ravaud C, Masmanian E, Weinberger M, Mayes S, Lemieux ME, Barnette DN, Gunadasa-Rohling M, Williams RM, Greaves DR, Trinh LA, Fraser SE, Dallas SL, Choudhury RP, Sauka-Spengler T, Riley PR. Macrophages directly contribute collagen to scar formation during zebrafish heart regeneration and mouse heart repair. Nat Commun 11: 600, 2020. doi:10.1038/s41467-019-14263-2. 32001677 PMC6992796

[20] Wiig H, Keskin D, Kalluri R. Interaction between the extracellular matrix and lymphatics: consequences for lymphangiogenesis and lymphatic function. Matrix Biol 29: 645–656, 2010. doi:10.1016/j.matbio.2010.08.001. 20727409 PMC3992865

[21] Hong YK, Lange-Asschenfeldt B, Velasco P, Hirakawa S, Kunstfeld R, Brown LF, Bohlen P, Senger DR, Detmar M. VEGF-A promotes tissue repair-associated lymphatic vessel formation via VEGFR-2 and the alpha1beta1 and alpha2beta1 integrins. FASEB J 18: 1111–1113, 2004. doi:10.1096/fj.03-1179fje. 15132990

[22] Hogan BM, Bos FL, Bussmann J, Witte M, Chi NC, Duckers HJ, Schulte-Merker S. Ccbe1 is required for embryonic lymphangiogenesis and venous sprouting. Nat Genet 41: 396–398, 2009. doi:10.1038/ng.321. 19287381

[23] Mitsuhashi A, Koyama K, Ogino H, Afroj T, Nguyen NT, Yoneda H, Otsuka K, Sugimoto M, Kondoh O, Nokihara H, Hanibuchi M, Takizawa H, Shinohara T, Nishioka Y. Identification of fibrocyte cluster in tumors reveals the role in antitumor immunity by PD-L1 blockade. Cell Rep 42: 112162, 2023. doi:10.1016/j.celrep.2023.112162. 36870329

